# Glucocorticoid-Dependent Mechanisms of Brain Tolerance to Hypoxia

**DOI:** 10.3390/ijms22157982

**Published:** 2021-07-26

**Authors:** Elena Rybnikova, Natalia Nalivaeva

**Affiliations:** 1 I. P. Pavlov Institute of Physiology, Russian Academy of Sciences, 199034 Saint Petersburg, Russia; 2 I. M. Sechenov Institute of Evolutionary Physiology and Biochemistry, Russian Academy of Sciences, 194223 Saint Petersburg, Russia; natalia.nalivaeva@iephb.ru

**Keywords:** glucocorticoids, glucocorticoid receptors, hypoxia, hypoxic preconditioning, hypoxic tolerance, HIF-1, prenatal hypoxia

## Abstract

Adaptation of organisms to stressors is coordinated by the hypothalamic-pituitary-adrenal axis (HPA), which involves glucocorticoids (GCs) and glucocorticoid receptors (GRs). Although the effects of GCs are well characterized, their impact on brain adaptation to hypoxia/ischemia is still understudied. The brain is not only the most susceptible to hypoxic injury, but also vulnerable to GC-induced damage, which makes studying the mechanisms of brain hypoxic tolerance and resistance to stress-related elevation of GCs of great importance. Cross-talk between the molecular mechanisms activated in neuronal cells by hypoxia and GCs provides a platform for developing the most effective and safe means for prevention and treatment of hypoxia-induced brain damage, including hypoxic pre- and post-conditioning. Taking into account that hypoxia- and GC-induced reprogramming significantly affects the development of organisms during embryogenesis, studies of the effects of prenatal and neonatal hypoxia on health in later life are of particular interest. This mini review discusses the accumulated data on the dynamics of the HPA activation in injurious and non-injurious hypoxia, the role of the brain GRs in these processes, interaction of GCs and hypoxia-inducible factor HIF-1, as well as cross-talk between GC and hypoxic signaling. It also identifies underdeveloped areas and suggests directions for further prospective studies.

## 1. Introduction

Survival of organisms requires continuous responsiveness and adaptation to various external stimuli and fluctuations of the internal environment. This is achieved through a set of physiological reactions and biochemical changes induced and coordinated by the major system of adaptation—the hypothalamic pituitary adrenal (HPA) axis [1,2], and particularly by glucocorticoids (GCs) as their effector hormones. GCs are released by the adrenal cortex in response to stressful environmental changes. They act both on peripheral organs and the brain, affecting their metabolism, immunity, neurotransmission, excitotoxicity, neuroplasticity, behavior, emotions and cognition [3]. It is impossible to find a physiological process that is not directly or indirectly affected by GCs, but nevertheless the impact of these hormones on brain adaptation to hypoxia/ischemia and development of hypoxic/ischemic tolerance is still understudied.

Pathological conditions of a hypoxic nature are among the most prevalent, taking place in both the internal and external environments. They are often associated with stroke, ischemia or other cardiovascular pathologies. Various organs and tissues have different resistance to hypoxia, but the brain is the most susceptible to hypoxic injury, similar to GC-induced injury. For this reason, studying the mechanisms whereby stimulation might increase brain resistance to hypoxia and result in formation of brain hypoxic tolerance is a very important scientific problem. Extensive research has been performed to uncover the impact of apoptosis-related factors, intracellular cascades, transcription factors, activation of late response genes and de novo protein synthesis in response to hypoxia (for review see [4,5]).

Compelling data demonstrate the protective effects of GCs against ischemia-reperfusion or hypoxic injury in different organs [6,7,8], including the brain [4,9,10]. Administration of GCs results in an increased brain tolerance against hypoxia, and their efficacy in some cases is even higher than that of ischemic preconditioning [11]. Numerous studies also demonstrate high efficacy of GCs in increasing brain tolerance to hypobaric hypoxia at high altitudes, and so far dexamethasone remains the best choice to prevent and treat acute mountain sickness and high altitude illness, including high altitude brain edema [12]. Moreover, the protective effect of dexamethasone in the recent coronavirus disease (COVID-19) suggests that high-altitude residence could be also beneficial for the disease outcome [13]. These facts allow us to hypothesize that GC-dependent mechanisms might contribute to the formation of cerebral hypoxic/ischemic tolerance induced by hypoxic/ischemic preconditioning. This was indeed supported by Sharp and colleagues [14], who analyzed the whole genome changes in response to “hypoxia preconditioning” and found that 17 of 70 verified HIF-1 target genes and five genes of GC signaling (NFKBIA, FKBP5, CDKN1A, TSC22D3, SGK1) were activated.

However, very little is yet known about the mechanisms through which GCs contribute to the induction of hypoxic/ischemic tolerance and interact with specific molecular mechanisms of cellular adaptations to hypoxia, particularly in such vulnerable cells as brain neurons. The aim of the present mini-review is to discuss the evidence accumulated to date and to identify blank areas or hotspots for further prospective studies.

## 2. Glucocorticoids and Hypoxia-Brain Tolerance and Cross-Talk Mechanisms

### 2.1. Dynamics of the HPA Activation in Injurious and Non-Injurious Hypoxia

**Figure 1:**
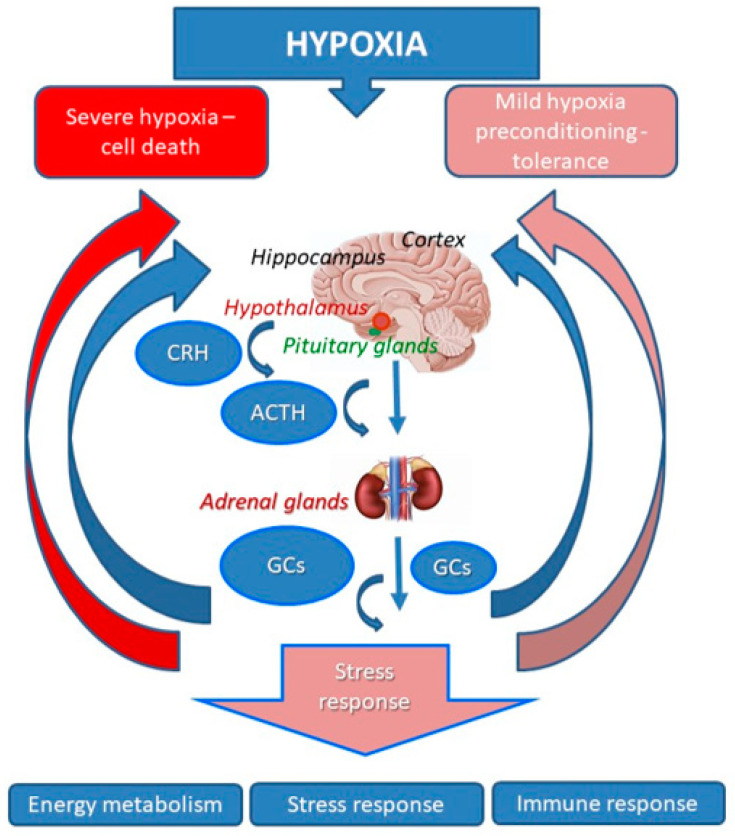
Schematic representation of HPA and glucocorticoid response to hypoxia and hypoxic pre-conditioning. Hypoxia induces the hypothalamus to produce and release corticotropin-releasing hormone (CRH), under whose action the anterior pituitary gland releases a number of stress-related hormones, including the adrenocorticotropic hormone (ACTH). ACTH stimulates cells of the adrenal glands to produce and release the GC stress hormone cortisol (corticosterone). GCs, via a feed-back mechanism, control the release of CRH and ACTH, and also affect metabolism of other neuronal cells. Severe hypoxia results in elevated levels of GCs resulting in neuroinflammation and neuronal cell death. On the other hand, pre-conditioning to mild hypoxia attenuates GC levels, resulting in development of neuronal cell adaptive mechanisms and brain tolerance to further severe hypoxia episodes or effects of other stressors.

At the level of the organism, a hypoxic challenge is perceived as a non-specific stressor and therefore a specific dynamic of the HPA response. This results in mobilization of the molecular resources necessary to adapt to the hypoxic factor. According to our earlier reports, severe injurious hypoxia induced moderate activation of the HPA with gradually escalating (up to 24 h) serum corticosterone levels. Such dynamics represent a maladaptive mode with sharp attenuation of the HPA reactivity and insufficiency of the glucocorticoid feedback [9]. A similar elongated phase of the HPA activation was also described for other brain pathologies (e.g., following traumatic brain injury (TBI) in animal models [15] and patients [16]. A clinical study showed that, following severe TBI, the baseline serum cortisol levels decreased during three days after TBI, and as such suppressed the HPA axis activation resulting in a worsened outcome [16]. Other studies have confirmed that the HPA axis suppression contributes to the compromised recovery, worsened long-term outcomes and development of disabilities after TBI and ischemic stroke [17,18], while treatment with synthetic GCs significantly improved rehabilitation. However, using exogenous GCs to compensate for the HPA insufficiency cannot be considered as a good therapeutic strategy, since the mechanisms of the HPA regulation remain impaired, and exogenous GCs given in this background can even aggravate the violation of the feedback mechanisms and lead to glucocorticoid resistance. For this purpose, the most promising direction could be activation of the HPA at the neuroendocrine level, although until such approaches are developed, it is advisable to use non-pharmacological methods that also optimize functions of the HPA. In particular, some pre-conditioning and post-conditioning techniques might be effectively and safely used to correct the malfunctioning HPA in post-hypoxic states. Indeed, our studies have shown that application of neuroprotective pre-conditioning before severe hypoxia resulted in reprogramming of the HPA response to severe hypoxia. The pre-conditioning technique consisted of three episodes of hypobaric hypoxia (360 mm Hg for 2 h spaced at 24 h) restoring the biphasic mode of HPA response with rapid activation and proper feedback inhibition of GC release [9,19]. The same effect on HPA was observed when three episodes of mild hypobaric hypoxia were given, following severe hypoxic insult, as the post-conditioning treatment [20]. Apparently, reprogramming of the HPA contributes significantly to the development of hypoxic tolerance induced by hypoxic pre-and post-conditioning. In contrast to severe injurious hypoxia, mild hypoxic episodes, used in our study for pre-and post-conditioning, themselves produced moderate biphasic HPA activation, which confirms its pro-adaptive mode (Figure 1).

### 2.2. Glucocorticoid Receptors of the Brain

The receptors of GCs—corticosteroid receptors—are ligand-dependent transcription factors. Inactive corticosteroid receptors are located in the cytosol as multi-protein complexes with several molecular chaperones [21]. Ligand binding releases the receptor from this complex, allowing its translocation to the nucleus, where it binds to the specific DNA regions (glucocorticoid-response elements, GRE) of hundreds of target genes, either activating or repressing them [22,23,24,25]. Two subtypes of corticosteroid receptors have been identified, the mineralocorticoid receptor (MR) and the glucocorticoid receptor (GR) [26]. Since MR has a 10-fold higher affinity to GCs than GRs [27], they appear to act mainly at GC baseline levels and therefore are responsible for the HPA functioning in normal, non-stressful conditions. In contrast, GRs are functionally active at stress-induced high GC levels and therefore orchestrate stress responses of the cells, organs, and the whole body. One of the main and most vulnerable targets of GCs is the brain, where excessive or chronic activation of GRs may be detrimental for neuronal function and survival [28,29].

It could be assumed that GRs, acting at high GC concentrations, might be more functionally relevant to adaptive neuronal responses to hypoxia and mechanisms contributing to hypoxic tolerance than MRs. Besides this, the molecular mechanisms by which GCs regulate gene expression have been better studied for GRs than for MRs. Taking this into account, herein we mainly focus on the GR and role of GCs in response to hypoxia, representing a potent stressor that activates the HPA axis. The central role in inducing the overall adaptive response has been ascribed to the hypoxia-inducible factor-1 (HIF-1), which is the main and most ancient regulator of cellular adaptation to hypoxia. Taking into consideration the roles of HIF-1 in adaptation to hypoxia and the universal pro-adaptive action of GCs, certain close interactions of these two factors could be suggested, but so far this has not yet been proven directly. Below, we review some data supporting this hypothesis.

### 2.3. Interaction of GCs and Hypoxia-Inducible Factor HIF-1

HIF1 is a transcription factor, which is a heterodimer of the proteins HIF-1α and HIF-1β, which have a DNA binding domain, containing a central PAS (Per-ARNT-Sim) domain that provides heterodimerization and N-and C-terminal transactivator domains that mediate the launch of transcription [30,31]. While HIF-1β is constitutively expressed at a steady level, HIF-1α, being a regulatory subunit, is accumulated only under hypoxic conditions, [30]. In the presence of oxygen, HIF-1α is hydroxylated by a prolyl hydroxylase, ubiquitinylated, and undergoes proteasomal degradation. Under hypoxic conditions, due to the absence of an oxidant, the prolyl hydroxylase is not active and the ubiquitin ligase VHL does not recognize HIF-1α, leading to its accumulation, dimerization with HIF-1β and translocation to the nucleus. In the nucleus, the HIF-1 heterodimer binds to hypoxia-responsive elements (HRE) on gene promoters, activating the transcription of target genes, including vascular endothelial growth factor (VEGF), glucose transporter 1 (GLUT1), enzymes of the glycolysis pathway, lactate dehydrogenase, erythropoietin, and many other genes necessary to provide a comprehensive program of adaptation to hypoxia [30,31,32,33,34,35,36]. HIF-1 appears to be involved in the up-regulation of glycolysis in astrocytes [37] as well as in the Schwann cells [38]. In neuronal cells HIF-1-dependent activation of glycolysis was shown to be involved in the process of lipid synthesis required for neurite growth [39]. GCs are also known to regulate glucose metabolism of the brain in multiple steps [40] but there is no evidence yet on their cross-talk with HIF-1 in these processes.

The involvement of HIF-1 in the development of hypoxic/ischemic tolerance of the brain has been well-documented in the studies with different types of hypoxic/ischemic pre- and post-conditioning [41,42,43]. We have investigated the role of HIF-1 using our animal model of pre-conditioning, by repetitive mild hypobaric hypoxia and assessing the HPA axis functioning. It was found that induction of brain hypoxic tolerance by pre-and post-conditioning was associated with two major events: an up-regulation of immediate HIF-1α expression [9,44] and potentiation of GC release in response to injurious exposures [9,19]. A number of recent comparative studies analyzing the effective (three-trial) and non-effective (one-trial or six-trial) pre-conditioning modes allowed us to suggest that both increased HIF-1α expression and potentiation of GC release are necessary for effective pre-conditioning [9,45,46]. In addition, blocking either of these by injection of the HIF-1 translation suppressor topotecan [47] or the GC synthesis inhibitor metyrapone [8] abolished the protective effects of hypoxic/ischemic pre-conditioning.

Despite numerous convincing proofs of the neuroprotective role of HIF-1 [48,49,50], our recent finding that pharmacologic suppression of HIF-1α translation by injection of topotecan prevented neuronal apoptosis caused by severe hypoxia indicates a dual role of HIF-1 [51]. Another finding supporting this assumption was reported by Kirova and colleagues [52], showing that innate cerebral resistance to hypoxia in rats correlated inversely with HIF-1α levels. While the basal HIF-1α expression in neurons of low-resistant rats was higher compared to high-resistant animals, it correlated with more pronounced neuronal injury after focal ischemia in their prefrontal cortex [52]. Moreover, inactivation of HIF-1α was found to be neuroprotective by reducing the severity of TBI-induced brain injury, by activation of microglia and NLRP3 inflammasome-mediated pyroptosis [53]. The effect of HIF-1 activation under hypoxia was shown to be cell-specific, since loss of HIF-1α function in neurons reduced neuronal viability while selective loss of HIF-1 in astrocytes protected neurons from cell death caused by hypoxia [54]. The question about possible dual action of HIF-1, which requires special attention, makes the overall picture even more complex.

During the last two decades, numerous reports on increased cortisol levels either at high altitude or intrauterine hypoxia, or protective effects of dexamethasone against acute mountain sickness (for review see [12]) led to the assumption that there is a functionally significant cross-talk between GCs and adaptation to hypoxia. In this regard, it has been demonstrated that in hypoxic conditions GCs enhance expression of the hypoxia-responsive genes, such as VEGF, glucose transporters and adrenomedullin in various cell cultures [55]. Importantly, the enhancement of their transcription was seen exclusively in GR-transfected cells, not in MR-transfected cells. In contrast, an inhibitory action of GCs on HIF-1 dependent gene expression was also reported [56,57]. Thus, in human hepatoma cells, it was shown that dexamethasone treatment increased HIF-1α levels in the cytosol but decreased nuclear HIF-1α levels and HIF-1 binding to DNA, which resulted in suppression of hypoxia-dependent expression of the HIF-1 target gene VEGF. This effect was not observed in the cells lacking GRs [56]. In human pulmonary epithelial A549 cells, dexamethasone inhibited hypoxia-induced activation of COX-2 expression [57].

Kodama and colleagues [55] hypothesized that some direct protein–protein interactions between the GR and HIF-1 might be the underlying biochemical mechanism for GC-dependent up-regulation of HIF-1 target genes. However, they failed to confirm it by GST pull-down assay, although they clearly showed a colocalization of the GR and HIF-1. Nevertheless, they have demonstrated that the effect of GCs on HIF-1 target genes requires ligand-bound conformation of the GR ligand-binding domain, which is necessary to recruit cofactors including transcriptional coactivators [55]. Based on their data, Kodama and colleagues [55] suggested that the C-terminal half of HIF-1 is a target of the GRs, and that the GRs might be recruited to a multiprotein complex including HIF-1 and probably another yet unidentified factor. As such, further studies are needed to uncover the exact mechanisms and prove this suggestion experimentally. However, it is already obvious that there is an interplay between GCs and HIF-1 at the level of GRs [58].

In theory, GRs can interact with HIF-1 at multiple levels, starting from regulation of each other’s gene expressions up to their interaction in the cytosol. The latter has been described in the liver of zebrafish mutant lines and human hepatocytes in vitro. This process was independent of GR DNA binding, since GCs promoted degradation of Von Hippel Lindau protein, followed by HIF-1α stabilization and activation of HIF-targeted transcriptional responses [59]. Whether this effect is tissue-specific or universal remains unclear. However, our studies in animal models of depression provided indirect evidence of non-specificity of such a mechanism of HIF-1 activation for the liver. Specifically, we have reported that development of depressive-like stress-related pathology in rat models was accompanied by persistent up-regulation of HIF-1α from the 5th to 10th day after psychoemotional stress [60]. This was a surprising finding because no hypoxia was induced in these experiments, implying that some oxygen-independent mechanisms of HIF-1 activation were involved. The findings of Vettori and colleagues cited above [59] added to our understanding that such a mechanism can be associated with stabilization of HIF-1α by GCs which are chronically elevated in depression. Whether such long-lasting activation of HIF-1 by high GC levels in depression represents a side-effect or is of innate nature requires, in our opinion, special attention. Such activation can contribute undesirably to the disturbances of the metabolic processes and vascular growth in the depressed organism, and thereby worsens the disease state.

A recent study by Marchi and colleagues [61] in zebrafish larvae demonstrated that the up-regulation of HIF-1 signaling repressed both the responsiveness of GR and cortisol levels, whereas GCs enhanced the activity of HIF-1. This effect was mediated by both GR and MR receptor subtypes, and the authors suggested that GCs promote HIF-1 signaling via multiple routes [61].

Our studies also indicated the involvement of both GR and MR in the induction of brain hypoxic tolerance. The effective mode of hypoxic preconditioning (three-trial) used in our studies, in contrast to the non-effective (one-or six-trial) ones, produced a specific pattern of balanced hippocampal GR and MR expression before and after severe injurious hypoxia [62]. These changes obviously contributed to the molecular and neuroendocrine mechanisms of hypoxic tolerance, since in addition to the protection of neurons they also ensured proper functioning of the glucocorticoid feedback regulation of the HPA axis that is extremely important in harmful or stressful conditions.

### 2.4. A Cross-Talk between Glucocorticoid and Hypoxic Signaling

**Figure 2:**
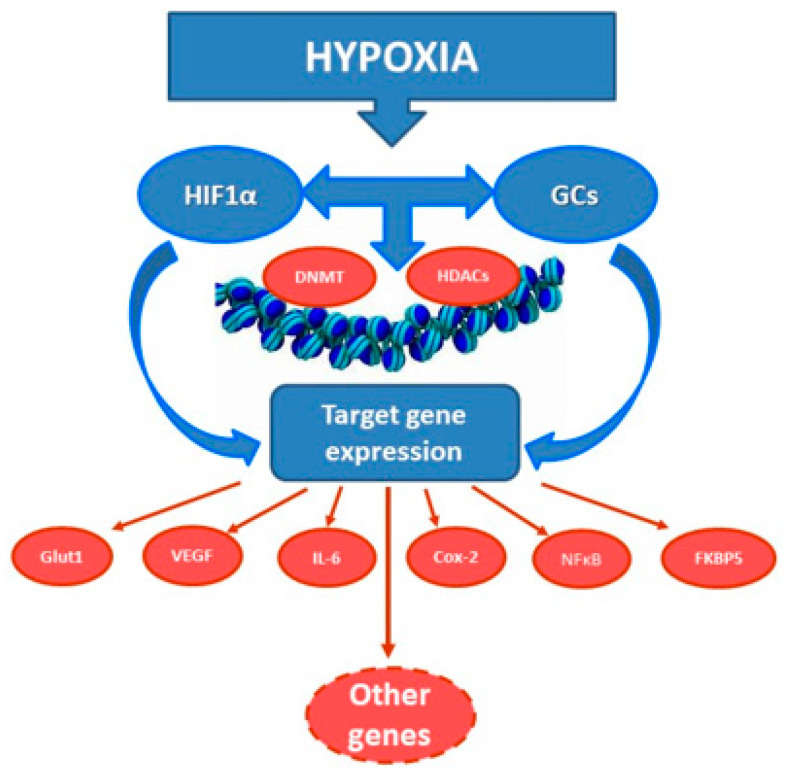
Hypoxia-induced increase of GC and HIF-1α levels facilitates their intracellular cross-talk involving related downstream mechanisms or directly leads to activation of a number of their target genes. It might also reprogram expression of other genes via modulation of the activity of DNA methyltransferases and histone deacetylases, leading to modification of chromatin structure and accessibility of gene promoters to various transcription or regulatory factors.

In addition to the direct interaction of GRs and HIF-1, a cross-talk between the components of their downstream signaling cascades can also be suggested. Although the existence of such cross-talk in the brain still has to be proven, some data obtained in non-neuronal cells implies that the interactions between the signaling cascades activated by GCs and hypoxia in fact exist, and can be either synergistic or reciprocal (Figure 2). Indeed, GCs were shown to inhibit expression of an important HIF-1 target, COX-2, in human epithelial pulmonary cells subjected to hypoxia [57]. The mechanism involved induction of an anti-inflammatory glucocorticoid-induced leucine zipper (GILZ), which suppressed expression of HIF-1α at the protein level, and thereby affected its down-stream signaling pathway.

Both hypoxia and dexamethasone independently induced and co-regulated expression and activation of the guanosine triphosphate (GTP)-binding protein RhoB, an important stress sensor which contributes to the regulation of cytoskeletal organization, cell proliferation and survival [63]. HIF-1α, JNK and ERK were also shown to be involved in up-regulation of RhoB in hypoxia [64].

GR may also regulate transcription of the target genes without direct binding to DNA, e.g., by modulating the activity of other transcription factors through protein–protein interactions [65]. These interactions could provide another platform for the cross-talk with hypoxia-driven specific mechanisms including HIF-1. In such a manner, GRs reciprocally interfere with the Jun amino-terminal kinase (JNK) signal transduction pathway [66] blocking JNK activity. On the other hand, we have demonstrated that inactivation of the JNK pathway contributes to the development of pre-conditioning-induced brain tolerance [67]. Obviously, these data indirectly support the hypothesis of possible non-genomic interactions of GCs and the components of hypoxic signaling in regulating the activity of transcription factors, but this needs further investigation.

### 2.5. Glucocorticoids, Hypoxia and Inflammation

Triggering neuroinflammatory reactions in the brain is an important component of the ischemic cascade contributing to exacerbation of the injury [68]. Accordingly, well-documented therapeutic effects of GCs in hypoxic/ischemic states are commonly attributed to their immunosuppressive and anti-inflammatory actions [69] which is especially relevant to various pathological conditions in the brain [28,29]. It was shown that hypoxia and GC signaling converge on the promoter site of the proinflammatory factors to regulate reciprocally their expression in a T-lymphocyte cell line model [70]. Huang and colleagues [71] showed that hypoxia reduced the GRα (but not GRβ) expression and attenuated the anti-inflammatory action of GCs in human alveolar epithelial cells, whereas others have reported that the GR agonist dexamethasone impairs the pro-inflammatory action of HIF-1 [56]. Regarding the regulation of inflammation, special attention should be paid to GILZ, which is thought to be an important mediator of anti-inflammatory and immune-suppressive actions of GCs. Wang and colleagues reported that hypoxia itself not only remarkably up-regulated expression of GILZ, but also significantly enhanced GC-induced expression of GILZ in macrophages and the spleen of rats [72]. Hypoxia-induced up-regulation of GILZ also involved ERK activation. Inhibition of GILZ activation resulted in a significant increase in mRNA production and protein secretion of IL-1β and IL-6 and abrogated the inhibitory effect of GCs on expression of IL-1β and IL-6 in hypoxia. These findings suggest that GILZ is importantly involved in adjusting adaptive responses to hypoxia both by down-regulation of the pro-inflammatory processes and mediation of the anti-inflammatory action of GCs under hypoxic conditions.

GGs can also act at the level of the blood–brain barrier (BBB). There are data indicating that after treatment with dexamethasone, immortalized mouse brain endothelial cells injured by in vitro blast injury had improved trans-endothelial electric resistance recovery and increased tight junction ZO-1 immunostaining compared to untreated cells [73]. This suggests that GCs might play an important role in BBB recovery after TBI, and in the decrease of neuro-inflammation by preventing peripheral immune cells from migrating into the brain. This anti-inflammatory effect of GCs is believed to be maintained through GR-mediated activation of GREs, which results in suppressed mRNA expression of pro-inflammatory cytokines including IL1β, TNFα, and IL-6, and enhanced expression of anti-inflammatory cytokines IL-10 and TGFβ [74]. In a mouse model of TBI, it was also demonstrated that combination treatment with dexamethasone and melatonin had beneficial synergistic effects on the infarct area and volume, and on the density of blood vessels in the brain sections affected by the trauma [75]. Since BBB integrity is compromised in various CNS disorders, GC treatment aiming to improve its tightness has been considered as a mainstream treatment in such diseases as stroke, multiple sclerosis, HIV-1-associated dementia, Alzheimer’s disease, and cerebral malaria (for review see [76]). Although GCs have significant systemic side effects, their use for treatment of peritumoral brain edema caused by BBB breakdown is still one of the main approaches in therapy for brain tumors. The therapeutic effect of GCs in this case might occur via reducing tumor cell viability and suppressing VEGF production in tumor cells. Moreover, GCs can modulate expression and distribution of tight junction protein occluding, claudin-5, and ZO-1 in endothelial cells [77].

### 2.6. Effects of Prenatal Hypoxia on HPA, GC and Hypoxic Response in Later Life

Prenatal and neonatal hypoxia, being the most common complications of pregnancy and labor, increase significantly the risk of the development of various metabolic and neurological disorders in later life [78,79]. The concept of fetal or developmental origins of diseases is now well accepted, but the mechanisms underlying this “programming” are poorly understood. However, it is becoming evident that glucocorticoid effects and epigenetic changes play an important part in these processes [80,81]. Although the fetus has a high capacity to respond to stress during development mediated in part by the HPA axis (reviewed in [82]), hypoxia can have a significant impact on the fetal HPA axis itself, resulting in its malfunctioning during embryonic development and after birth [83]. On the other hand, HIF1α, although mediating the responses to abnormal hypoxic exposure, is also necessary for normal fetal development [84,85]. Its absence in mice leads to abnormalities in CNS development and embryonic death by E11 [86]. Although HIF-1 overactivation had no effect on cerebellar cells, it was shown to prime both cerebellar granule neuron precursors and Purkinje cells for injury via glucocorticoids, suggesting that hypoxia and HIF-1 activation, together with postnatal glucocorticoid administration via different pathways, contributes to cerebellar injury [87]. Because of the lack of systematic studies, the question of whether the role of HIF-1α in CNS development is related to physiological hypoxia, or whether this is a hypoxia-independent function, is still far from understood, although there are attempts to correlate these events in relation to the formation of brain connectivity [88].

Prenatal severe hypoxia (PSH) was shown to result in continuously elevated baseline corticosterone blood levels in the adult and aged rats [89] accompanied by a progressive deficit of GR expression in the liver. Elevated corticosterone levels also resulted in impaired glucocorticoid regulation mechanisms in the hippocampus of new-born rat pups, which persisted throughout their life. Although negative effects of PSH could be prevented to some extent by treatment of pregnant females undergoing hypoxia with an inhibitor of corticosterone synthesis, metyrapone [89], the consequences of such interventions to the HPA regulatory status of the developing organism cannot be underestimated. Prenatal hypoxia was also found to lead to age and region-dependent changes in HIF-1α, VEGF and GR, resulting in anxiety-like behavior in three-month-old mice [90]. Although these authors reported an increase in GR protein levels in brain structures, another study demonstrated that maternal hypoxia decreased both GR mRNA and protein levels in the fetal brain and neonatal hippocampus, as well as abolishing the dexamethasone-mediated neuroprotective effect in pup brains [91]. This decreased GR expression correlated with increased DNA methylation and decreased binding of transcription factors Egr-1 and Sp1 to the GR gene exon 17 and 111 promoters, resulting in their reduced expression of GR exon 17 and 111 mRNA variants.

Prenatal hypoxia was also shown to induce epigenetic repression of the GR gene in the developing heart, resulting in significantly reduced levels of GR mRNA and protein in the hearts of adult (five months old) rat male offspring, but not in females due to the differential changes of alternative exon1 mRNA variants of the GR gene in male and female hearts [80]. This decreased GR expression in the heart of male individuals correlated with increased methylation of CpG dinucleotides in GR gene promoters, resulting in reduced binding of transcription factors which might increase the vulnerability of male individuals subjected to prenatal hypoxia to development of heart pathology, including ischemic heart disease. Treatment of neonatal hypoxic rats with a DNA-hypomethylating agent 5-aza-2’-deoxycytidine was found to reverse hypoxia-induced promoter methylation and restored GR expression, which had a protective effect against ischemia and reperfusion injury in the hearts of offspring at the age of one month [92]. These findings demonstrate that prenatal hypoxia can induce epigenetic repression of the GR gene via DNA methylation-dependent mechanisms which can be taken into account when designing therapeutic strategies for preventing development of ischemic heart disease in male individuals.

There are also indications that downregulation of GR gene expression in the heart caused by prenatal hypoxia might involve miR-210, whose levels were shown to increase in response to hypoxia in fetal rat cardiomyocytes, and that this miR-210 increase was HIF-1α-dependent [93]. Increased miR-210 levels with subsequent downregulation of GR were also demonstrated in the brain of 10-day-old rat pups submitted to perinatal hypoxia-ischemia (HI), who developed significant encephalopathy, while intranasal administration to these animals of miR-210 inhibiting complementary locked nucleic acid oligonucleotides (miR-210-LNA) significantly reversed HI-induced neuronal death and improved long-term neurological recovery [94]. These studies add a new dimension to our understanding of the mechanisms underlying prenatal hypoxia-induced changes in GC-related mechanisms of animal and human postnatal development.

The fact that some changes in expression of neuronal genes in rat pups subjected to prenatal hypoxia could be attenuated by administration of a histone-deacetylase inhibitor, valproic acid [95,96,97], suggests that prenatal hypoxia might also affect gene expression in the developing organism via histone modification mechanisms. Moreover, activity of some HDACs (in particular of lysine histone deacetylase KDAC1) are involved in GR-mediated activation of GR target genes, and as such their modification caused by prenatal hypoxia may result in impaired GR-signaling in the developing organism [98].

Pre-conditioning to mild hypoxia during pregnancy was shown to have beneficial effects both at the survival level and neuroprotection in rat pups subjected to perinatal stroke, which was abolished by iNOS inhibition [99]. In a chick embryo model, beneficial effects of hypoxic pre-conditioning were shown to be HIF-1 dependent, increasing developing organism tolerance to future hypoxic insults i [100]. Some authors even consider pregnancy to be an important period to increase brain tolerance to hypoxia-ischemia insults in later life [101]. However, administration of GCs for treatment of various adverse conditions during pregnancy involving fetal hypoxia, although in many cases beneficial, can result in adrenal developmental toxicity up to adulthood, which has recently been extensively reviewed [102].

**Figure 3:**
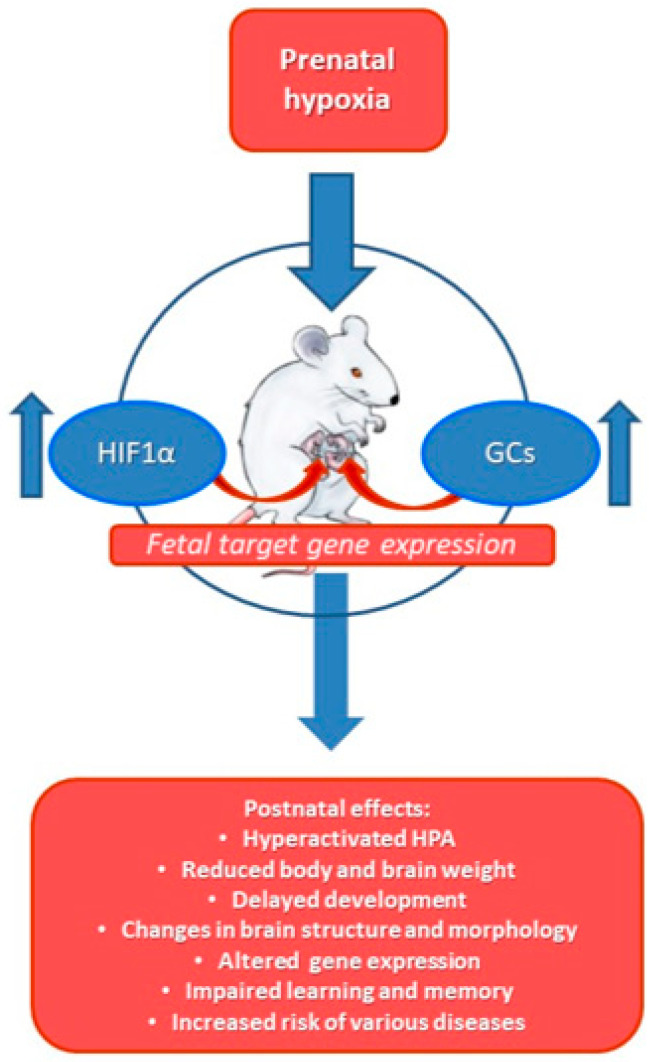
Prenatal hypoxia leading to changes both in the maternal and fetal HPA results in increased levels of GCs and HIF-1α in the developing fetus. This will lead to changes in expression of a number of fetal genes, and affects the developmental program of the new-born organism, predisposing it to various pathologies in later life. It will also modify the response of various organs of the newborn to GCs, affecting their response to various stressors in later life.

To understand the molecular mechanisms involving normal and pathological hypoxic conditions and the role of HIF-1α, GR and their target gene activation for brain development in utero and in later life is of undeniable importance (Figure 3) and deserves significant research investment in the near future.

## 3. Summary

Development of cerebral hypoxic tolerance involves a reprogramming of the molecular and endocrine responses of cells and organs to hypoxic/ischemic injury. In particular, alterations of GR and MR expression in the brain, modification of intracellular GR signaling, potentiation of the HPA reactivity and feedback regulation appear to represent integral parts of such reprogramming. It results in beneficial conditions for optimal GC hypoxia-induced release and pro-adaptive action, including reinforcement of HIF-dependent pro-adaptive signaling, stimulation of anti-inflammatory defenses, and activation of non-specific adaptive resources of the whole organism.

## 4. Future Perspectives

Several promising directions for future research can be outlined. The most important problem to be solved is identification of possible side effects of HIF-1 in neuropathology. This is necessary for the introduction of restrictions on the use of HIF-1 activators in therapy that currently represent an ascending but risky trend. The second important question is to investigate GC-dependent activation of HIF-1 and its down-stream targets in various animal and human tissues, including the brain. Examining involvement of GILZ in suppression of neuroinflammation and development of brain hypoxic tolerance appears to represent another interesting area of research. As a practical result, these studies may lead to the design of a new generation of anti-inflammatory non-steroid drugs. Another fundamental problem with high social impact is clarification of possible pathogenic effects of HIF-1 in depression and other stress-related disorders, associated with HPA impairment. Such research might justify the benefits of using HIF-1 inhibitors in complex anti-depressant therapy, or the launching development of a novel type of anti-depressant drug. Finally, much research is required to understand the physiological significance of prenatal hypoxia for the development of the interplay between the HPA and organ responses to hypoxia, and its role in predisposition of the organism to development of various diseases in later life.
